# Evaluating Weaknesses of “Perceptual-Cognitive Training” and “Brain Training” Methods in Sport: An Ecological Dynamics Critique

**DOI:** 10.3389/fpsyg.2018.02468

**Published:** 2019-01-21

**Authors:** Ian Renshaw, Keith Davids, Duarte Araújo, Ana Lucas, William M. Roberts, Daniel J. Newcombe, Benjamin Franks

**Affiliations:** ^1^School of Exercise and Nutrition Sciences, Queensland University of Technology, Brisbane, QLD, Australia; ^2^Centre for Sports Engineering Research, Sheffield Hallam University, Sheffield, United Kingdom; ^3^CIPER, Faculdade de Motricidade Humana, Universidade de Lisboa, Lisbon, Portugal; ^4^School of Sport and Exercise, University of Gloucestershire, Cheltenham, United Kingdom; ^5^Department of Sport and Health Sciences, Oxford Brookes University, Oxford, United Kingdom; ^6^University Campus of Football Business, Wembley, United Kingdom

**Keywords:** perceptual-cognitive training, brain training, motor learning, neuroplasticity, ecological dynamics, sport performance

## Abstract

The recent upsurge in “brain training and perceptual-cognitive training,” proposing to improve isolated processes, such as brain function, visual perception, and decision-making, has created significant interest in elite sports practitioners, seeking to create an “edge” for athletes. The claims of these related “performance-enhancing industries” can be considered together as part of a *process training approach* proposing enhanced cognitive and perceptual skills and brain capacity to support performance in everyday life activities, including sport. For example, the “process training industry” promotes the idea that playing games not only makes you a better player but also makes you smarter, more alert, and a faster learner. In this position paper, we critically evaluate the effectiveness of both types of process training programmes in generalizing transfer to sport performance. These issues are addressed in three stages. First, we evaluate empirical evidence in support of perceptual-cognitive process training and its application to enhancing sport performance. Second, we critically review putative modularized mechanisms underpinning this kind of training, addressing limitations and subsequent problems. Specifically, we consider merits of this highly specific form of training, which focuses on training of isolated processes such as cognitive processes (attention, memory, thinking) and visual perception processes, separately from performance behaviors and actions. We conclude that these approaches may, at best, provide some “general transfer” of underlying processes to specific sport environments, but lack “specificity of transfer” to contextualize actual performance behaviors. A major weakness of process training methods is their focus on enhancing the performance in body “modules” (e.g., eye, brain, memory, anticipatory sub-systems). What is lacking is evidence on *how* these isolated components are modified and subsequently interact with other process “modules,” which are considered to underlie sport performance. Finally, we propose how an ecological dynamics approach, aligned with an embodied framework of cognition undermines the rationale that modularized processes can enhance performance in competitive sport. An ecological dynamics perspective proposes that the body is a complex adaptive system, interacting with performance environments in a functionally integrated manner, emphasizing that the inter-relation between motor processes, cognitive and perceptual functions, and the constraints of a sport task is best understood at the performer-environment scale of analysis.

## Introduction

There has been a recent upsurge in the “process training industry,” proposing how to improve isolated processes such as perceptual and cognitive capacities, like vision, attention, creative thinking, memory, “ultra-fast” decision-making, in order to improve performance at work, in tests and examinations, and sport. In related vein, a “brain training industry” also promotes the idea that, for example, playing digital games not only makes you better at playing these games but also makes you smarter, more alert, and helps you to learn faster. Brain training software presents neuroscience research about neuroplasticity to support the efficiency of their programs in training brain processes which are claimed to underpin performance effectiveness in many specific performance domains, including sport. Taken together, the claims of the perceptual-cognitive training and brain enhancing programs can be addressed under the rubric of “process training” industries. Their claims have created significant interest in elite sports practitioners, seeking to enhance athletic performance and create an “edge” for athletes. Process training industries claim that they can develop core abilities that underpin perceptual and cognitive skills and brain function beyond a particular sport. But does process training really improve perceptual-cognitive abilities and brain processes in a way transferable to sport tasks performance? Can this kind of training be used as a shortcut to enhance sport performance? In this position paper, we show how an ecological dynamics rationale can undermine the significance of these industry claims, focusing on the weakness of the supportive evidence on specificity of transfer of training.

While practice is essential to improving sports performance, the search for the so-called one-percenters is commonly promoted by leading sport scientists and practitioners who are seeking to create an “edge” or “marginal gains” for elite athletes. To that end, athletes spend significant periods in “off-field” training activities to enhance perceptual skills such as improving their visual search for information, maintaining attentional focus, and improving memory through cognitive skills training to build “knowledge” in support of their on-field performance. There are commercial interests driving the industrial scale of the financial value and promotion of these training devices/programmes in sport. Systematic reviews, such as that of Harris et al. (2018) clearly point to the industry worth billions of dollars behind the use of a range of different “process training devices/programmes” in sport. Their analysis shows that this “methodological approach” in sport has all the hallmark characteristics of an “industry.” Furthermore, these commercial interests are supported by the lucrative publication of popular science books, which have not necessarily been subject to rigorous peer review that academic literature has to undergo. Large swathes of the digital and conventional media provide broad support for the, sometimes, spurious claims of the process training industry (see Moreau et al., 2018).

Key questions for sport practitioners include: Is spending this amount of money justified? and What added value do these approaches purport to bring to performance? In this position paper, we address these questions and examine the evidence in support of these industry claims. We provide an ecological dynamics rationale to explain the limitations of the preferred modularized approach to training processes of perception and cognition and brain functions for understanding effects on sport performance. To address these issues, we first evaluate current approaches and evidence that support perceptual-cognitive training and its application in sport. We question the mechanisms purported to underpin process training and their limitations. A key focus is efficacy of theories of transfer, additive models, and evidence from neuroscience on brain plasticity (a key tenet for those advocating efficacy of “brain training”). In evaluating perceptual training effects, to exemplify our arguments, we provide an in-depth critical review of the evidence from the perspective of Quiet Eye, which could be considered as part of vision training programmes. We conclude by presenting an ecological dynamics rationale that proposes a context-dependent perspective on the role of cognition, perception, and action, highlighting that the human performer is a complex adaptive system, which interacts with performance environments in a functionally integrated manner.

A commonality in training programs for brain and perceptual-cognitive processes is that, currently, both industries tend to adopt a “modularized” approach. The assumption is that isolated processes (i.e., modules) in the brain and perceptual-cognitive functions can be trained separately from action in a performance context. Post-training, it is assumed that the enhanced process can be integrated back into the whole system with resultant performance duly enhanced. Indeed some proponents define CT as the act of improving what are termed “core cognitive processes,” which they assume to underlie sport performance (e.g., Walton et al., 2018). Substantial evidence for this claim is lacking, along with a rigorous definition of what is meant by the term “core cognitive processes.”

These assumptions in contemporary sport practice are based on the default approach of indirect perception underpinning sport psychologists’ attempts to describe and develop specific processes, such as perception, anticipation, attention, memory, and decision-making, by exposing performers to selectively adapt and modify displays such as still images, short video clips, and snapshots of performance environments (Araújo et al., 2017). This methodology is exemplified by schematic presentations of the position of chess pieces on a board (Chase and Simon, 1973), the co-positioning of players in two basketball teams or the serve actions of tennis players hitting topspin, slice, or flat serves (for a review, see Williams et al., 1999; Starkes et al., 2001). It does not seem to be considered important that an “action response” might constitute a button press in the studies evaluated (Walton et al., 2018). The assumption seems to be that any response will suffice to test effects of cognitive training on behavior, and it is unsurprising that a major outcome of current evaluations is a call for further investigation.

The assumptions underpinning the default approach in the literature supporting process training are not supported by other theoretical rationales, such as that of ecological dynamics (Araújo et al., 2017). In contrast, the ecological dynamics approach considers perceptual, cognition, and action sub-systems to be deeply intertwined in their activity, functioning as continuously integrated and highly coupled systems. Theoretically, it is not coherent and of little value to use a modularized approach and decouple processes of perception, cognition, and action to train them in isolation. Further, ecological dynamics is deeply concerned with knowledge and considers intentions and cognition to play an important role in theoretical explanations of human behavior (Davids et al., 2001a,b; Davids and Araújo, 2010a; Araújo et al., 2017). Determining how effective the indirect methods of developing underlying mechanisms of sports expertise is the key issue addressed in this paper. How can we enhance the cognition, perceptions, and actions through indirect means to support skilled performance that emerges through direct learning for athletes to become perceptually attuned to relevant properties of the environment? Here, we propose that effective interventions can be achieved by basing learning design on a view of knowledge, cognition, and intentions as deeply integrated and intertwined. Intentions, perception, and action interact to mutually constrain performance in practice and competition, and this key point needs to underpin the design of performance enrichment programs which target PC processes.

Training programs, based on indirect methods to build “knowledge about” the environment, enhance knowledge that can be used to describe (verbally or pictorially) performance. In contrast, the more direct “knowledge of” the environment (see Araújo et al., 2009; Araújo and Davids, 2011) supports how an individual interacts with a performance environment, intentionally, perceptually, and motorically, in picking up and utilizing *affordances* from the performance environment (defined as *opportunities for action* in ecological psychology). Gibson (1966, 1979) has suggested that *knowledge of* the environment is expressed by action and implies direct perception (i.e., the environment informs about what it is without the need of a mental—indirect—attribution of meaning) and direct experiences with specific environments. Adaptive behavior emerges as a continuous cycle where performers can prospectively control their actions by detecting information (Araújo et al., 2018). Consequently, ecological psychologists suggest that direct learning (Jacobs and Michaels, 2007) to develop “knowledge of” the environment is achieved by “doing.” Direct epistemological contact with an environment facilitates knowing how to achieve a task goal because it involves learning to detect and attune to key perceptual variables that regulate performance behaviors. Direct perception differs from indirect perception in its insistence of the mental integration of action, cognition, and perception through *active performance* to underpin human behavior. Ecological psychologists agree that knowledge could be obtained *via* mediated or indirect perception (Gibson, 1979) as a way of developing knowledge “second hand.” Essentially, the indirect acquisition of knowledge about the environment *via* a *passive* “classroom” approach, advocated and adopted in many contemporary approaches to sport psychology, is aligned with historical accounts of learning per se (i.e., *formal discipline theory*). Indirect knowledge about the environment involves shared knowledge about a performance environment mediated by language, symbols, pictures, displays, and verbal instructions (Araújo and Davids, 2011). The role of indirect forms of knowledge is to direct awareness and previous experiences for channeling a future “direct” experience with a specific environment (Reed, 1991). Here, we argue that, if enrichment programs are going to succeed in enhancing sport performance, they need to be predicated on the deeply intertwined relations between cognition (in the form of knowledge of the environment), actions, and perception, to pick up and utilize affordances during learning and performance.

These ideas are somewhat aligned with those in an embodied framework of cognition (e.g., Moreau et al., 2015) outlining the inter-relations between motor and cognitive processes, emphasizing that motor (cognitive) system involvement depends on specific cognitive (motor) interactions with a performance environment.

### Some Questions Over the Methods of the Process Training Industry

The recent upsurge in brain training programmes *via* computer “testing” has led to a multi-million GB pound industry (Owen et al., 2010), with proponents claiming improvements across the board in terms of cognitive functions for older people, preschoolers, and for those who play videogames, over those that do not. Brain training is appealing for consumers as it can be used outside of formal education and skill learning programmes, potentially marketing continuing cognitive development to a wider population. Despite the popularity, there remain some key questions that need to be addressed in future research.

#### What Are the Supportive Theory-Practice Links to Sustain General Ideas of Process Training?

Traditionally, perceptual-cognitive skills have been defined as the ability to identify and process environmental information, and integrate them with pre-existing knowledge and motor capabilities, to select and execute adequate actions (e.g., Marteniuk, 1976). In the 1960s and 1970s, there was an enormous amount of experimentation on “preprogramming” movements, muscle commands, the structure of motor programmes, central representations, attention and conscious control, movement execution in the absence of feedback, and invariant properties of abstract representations stored somewhere in the brain. This research led to disparate views of motor programmes in the literature, from an abstract, symbolic representation to a grouping of neuronal cells functioning in the vertebrate motor system The notion that skilled performance can be enhanced by storing motor programmes in the brain has had considerable influence on approaches to performance analysis and training in the sports sciences. For example, more recently, Summers and Anson (2009) revisited the notion of a motor programme, proposing that it was one of the most robust and durable phenomena in the motor control literature. An implicit assumption has been that skilled performance in sport is characterized by motor system invariance. This notion has led sports biomechanists to pursue the identification of an “ideal” movement template considered as a criterion of expert performance and acquired through numerous trial repetitions (e.g., Brisson and Alain, 1996). The implication is that motor programmes can be internalized in central nervous system structures of athletes with specific practice of a target movement assumed to be optimal with respect to time and learning (Gentile, 1972; Schöllhorn et al., 2006). Motor programmes reflect a traditional bias in psychology towards seeking personal attributions in explanations of human behavior and the neglect of situational attributions. This inherent bias in traditional psychology is exemplified by an overemphasis on the acquisition of *enriched internal states* in the brain (predicated on perceptual and cognitive skills) for explaining behavior regulation (Dunwoody, 2006; see also Davids and Araújo, 2010a,b; Araújo and Davids, 2011). The concept of *organismic asymmetry* refers to a predisposition to attribute behavior regulation solely to personal characteristics internalized in the brain by individuals through learning and practice, underplaying the role of the environment in transactions to support behavioral adaptation. Organismic asymmetry in traditional psychological theories reflects a preference for internal mechanisms, such as mental representations, to explain how the processes of perception, action, and cognition may be regulated. Dunwoody (2006) has expanded upon Brunswik’s (1955) criticisms of cognitive psychology explanations of behavior being biased away from person-environment interactions, as the basis of an “organismic asymmetry.” These theoretical biases and assumptions are harmonious with goals and aims of process training programmes based on learning to acquire a complex integrated representation of a movement in achieving expert performance in sport (Schmidt and Wrisberg, 2008).

Furthermore, some psychological theories have argued that it is the underlying cognitive control structures supporting performance that distinguish highly skilled individuals from their less-skilled counterparts (Abernethy et al., 2007). There is relevant research on the possible effectiveness of cognitive training in sport (Brown and Fletcher, 2017), specifically in interventions focusing on training perceptual-cognitive (P-C) skills such as pattern recognition, anticipation, decision-making, and quiet-eye (Farrow, 2013). Perceptual training programmes have been suggested as an additional aid to enhance performance preparation across all skill levels but are considered particularly useful for elite level performers who are time poor and have to conserve physical (energy) resources (Farrow, 2013) or avoid problems of overtraining and potential overuse injuries. However, while elite sports organizations may justify adopting such methods, it is somewhat surprising that few studies have examined the efficacy of such training programmes (Farrow, 2013). The same fundamental question underlies all process training programmes (i.e., the same concerns arise over general training programmes for enhancing brain processes and developing generic cognitive abilities): Do these programmes really improve cognitive abilities, perceptual skills, and/or brain processes in a way that is transferable to sport performance? Can this kind of training be used as a shortcut to enhance sport performance or are their perceived effects illusory?

Unsurprisingly, the majority of P-C training programmes have adopted similar methods to those used by researchers in measuring expertise, methods which have evolved in concert with emergent technologies. A clear tendency has been to use sports-specific content as a central feature of such training, as opposed to generalized training approaches, deemed as being ineffective (Abernethy and Wood, 2001). For example, early studies of expertise used static images of typical performance situations to examine cognitive and perceptual abilities of athletes, such as pattern recognition and recall skills (e.g., Chase and Simon, 1973; Allard and Starkes, 1980). Some researchers began to use temporal and spatial occlusion methods by requiring performers to watch dynamic video clips of “actions” of cricket bowlers, basketballers, footballers, squash, or badminton players, for example, in seeking to identify the information that novices and experts use to guide processes such as anticipation and decision-making. Many of these studies have recently been viewed as having a number of significant limitations including the use of small 2D screens, making information difficult to interpret; a lack of first person perspectives; and a putative “correct answer” associated with verbal or written responses instead of sport actions (van der Kamp et al., 2008).

#### How Strong Is Evidence for Some Claims of the Brain Training Industry?

Despite a large number of publications reporting tests of the effects of brain training interventions, evidence that training with commercial brain training software can enhance cognition, outside the laboratory tests is limited and inconsistent for performance in general (Simons et al., 2016) as well as in sport (Walton et al., 2018). For example, Owen et al. (2010) reported data from a six-week study in which 11,430 participants were trained online on cognitive tasks focusing on improving reasoning, memory, planning, visuospatial skills, and attention. Improvements were only registered in the cognitive tasks that were trained online. There was no evidence for transfer effects to untrained related tasks, even those considered to be “cognitively” closely related. Overall, it seems that practicing a cognitive task in brain training programs results in consistent improvements in performance *on that particular task* (near transfer). The available evidence that such training generalizes to other related tasks or to nondigital, ecological performance (far transfer) is not compelling (Simons et al., 2016).

Evidence on the limitations of brain training may not come as a surprise, given the plethora of research that has examined the underlying psychological processes underpinning expert sport performance, which involves a simultaneous participation of motor and cognitive processes (Williams and Ericsson, 2005).

#### What Does the Perceptual-Cognitive Training Industry Claim?

A systematic review by Harris et al. (2018) located 43 studies purporting to examine the beneficial effects of use of Commercial Cognitive Training devices on sport performance. Their search yielded only a single study that examined the most important issue of transfer effects to sport performance. Unsurprisingly, they concluded that there was limited evidence for transfer effects to sport performance. They attributed the lack of support for beneficial effects of perceptual-cognitive training to the current lack of studies seeking to provide evidence for these effects. There are two problems with this conclusion. First, it does not take into account that there may be many studies of perceptual-cognitive process training, which have not been submitted for publication because researchers did not find the expected benefits. This is a limitation that quantitative reviews always need to acknowledge, known as publication bias. Second, it is possible that the lack of beneficial effects may have been compounded by a lack of a substantive theoretical rationale implemented in research designs for how process training may yield benefits to performers. This is a weakness of contemporary research that we seek to address *via* this position statement.

#### What Can the Process Training Industry Learn From Research Seeking to Integrate Perception and Action in Sport Performance?

A key criticism of process training methods is that they do not allow participants to access both the dorsal and ventral visual cortical systems used in actual performances (van der Kamp et al., 2008). Developing technologies have enabled researchers more recently to undertake “*in situ*” studies of perception and action by using equipment like liquid occlusion goggles to enable more representative perception-action couplings to emerge during performance of a sport action. Ensuing data has revealed that requiring performers to utilize action-regulating perceptual information and demonstrate greater fidelity in perception-action responses may be more effective in highlighting expertise differences between athletes (e.g., Mann et al., 2010). Similar findings have been reported in eye tracking studies to assess visual search strategies. For example, goalkeepers were shown to alter their visual search patterns with respect to a “stimulus” presented and the action response required (Dicks et al., 2010; Dicks et al., 2017; Navia et al., 2017). Interestingly, the study by Dicks et al. (2010) demonstrated that the initiation of an action response by football goalkeepers facing penalties was mediated by their action capabilities. Goalkeepers who could dive “faster” were able to sample more of the penalty taker’s unfolding kick than those who moved more slowly. Pinder et al. (2011a,b) found that video training involving simulated cricket batting against a video-projected bowler on a “life-size” screen was partially representative of the fidelity of batting actions used against an actual bowler. When batting against the projected image, batters coupled the backswing of the bat and initial step, when preparing to get into position to hit the ball. However, the initiation of the downswing and swing velocity was different under the two conditions.

To enhance a tight coupling of perception and action systems during training in cricket, an ecological dynamics rationale proposes that batters need to couple the act of swinging a bat to hit a ball during actual flight, not an indirect image of a ball in flight simulated on a 2-dimensional video screen. The key issue is that the relevant affordances used by batters under the two conditions are different and quite specific. The implication is that extended practice in both different practice conditions is likely to lead to learners becoming more successful in batting *under those specific conditions*. The important question for cricket coaches (and of course skill acquisition theorists who advise them on learning design) is as follows: Which practice simulation is more closely related to the affordances available in cricket batting performance? To develop effective perception-action couplings in a time-efficient manner, the theoretical implication is that batters need to face real bowlers in practice, which would allow the batters to pick up and use affordances from the bowlers’ actions in delivering the ball (and earlier). To address issues faced by limited video training or use of ball projection machines, where no advanced information is available from opponents such as baseball pitchers or cricket bowlers, technologies such as ProBatter^TM^ have emerged, which seek to strengthen the links between perception and action. This has the potential to be a useful compromise, based on a powerful theoretical rationale in ecological dynamics, linking video images of a bowler’s actions with a ball projection machine. However, challenges emerge for participants when perceptual information provided in a video image is not representative of that provided by a bowler. In cricket bowling, bowlers change their bowling actions or their grips on the ball to deceive batters, imparting different spins, or to create swerve in ball flight. At present, projected ball flight with such technology does not reflect these important variations in flight. What you see is what you do not get. Additionally, the ball is projected through one hole and a batter can quickly become attuned to the information from the projection machine and learn to simply watch the projection hole only. Additionally, this fixed release point also limits the ability of the batter to determine the bounce point of the ball as a function of the angle of the bowler’s arm at ball release. The impact of practicing with these technological limitations on skill performance was demonstrated in a recent investigation combining video technology and a ball projection machine. Catching performance was negatively impacted with even a minor de-synchronization of perceptual images presented and flight characteristics of a ball projected by a machine (Stone et al., 2014).

Data such as these have important implications for those interested in designing and implementing perceptual training programmes. The evidence over the last 15 years from numerous reviews (e.g., Williams and Ward, 2007; Causer et al., 2012; Travassos et al., 2013; Vine et al., 2014; Broadbent et al., 2015; Slimani et al., 2016) is clear on the usefulness of P-C training. However, there is a major problem to be resolved. While P-C programmes “provide an idealized method for developing anticipation and decision-making judgments in athletes” (Broadbent et al., 2015, p. 329), the degree to which they transfer to competitive performance needs much more work. That is, transfer tests to competitive performance in sport settings are highly important and need to be implemented more frequently than they currently are in existing research (see also Harris et al., 2018). Overall, the current evidence is that P-C training effects remain specific to the confines of the training context: participants seem to improve at the training task. However, their effectiveness when transferred to sport performance is strongly mediated by the degree to which the training environment is *representative* of a performance environment and the *fidelity* of the actions required as a response (Travassos et al., 2013). To that end, a number of researchers have called for a more systematic programme of research to examine the nature and content of perceptual training approaches and their relationship with the skill of the user/learner (Farrow, 2013). Similarly, others have highlighted the need for such studies to be based on a strong theoretical framework that captures the complexity of cognition, perception, and action in sport performance and the nature of transfer from practice to performance (Seifert et al., 2013; Chow et al., in press).

#### Can We Be Sure That Research Findings on Use of P-C Skills Observed in Skilled Sport Performers Are Relevant for Training of Sub-elite Individuals?

One of the limitations of perceptual training programmes is that they often adopt a “one-size-fits all” approach in implying that the information used to anticipate and act in research studies is thought to be commonly used by all sport performers, regardless of skill level (Farrow, 2013). A good example where this approach has been adopted is in the research on Quiet Eye, which has recently seen a significant level of interest from researchers interested in P-C training but is now also attracting significant criticisms. The Quiet Eye (QE) phenomenon provides insights into gaze behaviors and their utility for decision-making and action in sport contexts (e.g., Vickers, 1996). QE, a consistent perceptual-cognitive measure investigated in sports research (cf. Mann et al., 2007; Baker and Wattie, 2016), is defined as the final fixation towards a specific location or object within 3* of visual angle or less for a minimum of 100 ms (Vickers, 2016) and has been described as process training (Wilson and Vine, 2018). The onset of QE occurs just before the critical movement of the action, while the offset occurs when the final fixation deviates from the located target for more than 100 ms (Panchuk and Vickers, 2006; Vickers, 2016). QE is proposed as one of the key determining factors associated with expert decision-making in sport, declared as *the* “perception-action variable” (Vickers, 2007; Causer et al., 2011). Rienhoff et al. (2016) meta-analysis located 581 published papers on QE research, evident of a significant amount of research activity over the years, which is almost exclusively situated within a linear cause-and-effect methodological landscape, based within a program dedicated to identifying a sole point of engagement with information within the perceptual field, typical of traditional decision-making studies (Glimcher, 2005; Chemero and Heyser, 2009). Further, it remains unclear why research on QE has been dominated by assumptions and terminology associated with an *information-processing* perspective towards cognition in sports performers (Michaels and Beek, 1995; Rienhoff et al., 2016). Regardless of this theoretical imbalance, some studies have utilized QE as a tool for perceptual training in sport. For example, QE training interventions have been used in attempts to train visual search strategies of nonexperts in similar tasks performed by expert counterparts. For example, Harle and Vickers (2001) study demonstrated the potential of QE-based training interventions, with significant improvements reported during free throw simulations, and notable fidelity of transfer into games (see also Causer et al., 2011).

While on the face of it, these data imply relevance of QE values which are universal for sport performers regardless of skill level, there have been numerous concerns raised over the legitimacy of QE training interventions. As Causer (2016, p2.) suggested in his commentary to Vickers (2016), “there are limited acquisition trials, short retention periods and multiple training interventions.” It is clear from the literature that the design of training interventions and research methods associated with them has been underdeveloped. For example, often trials are isolated incidents of performance, with the tasks being nonrepresentative of the constraints that exist in performance settings (Rienhoff et al., 2016). The lack of representative design is even more concerning when addressing dynamic team sports where there are numerous evolving landscapes governed by spatial and temporal constraints. The generalizability of findings in such studies to expert performance is currently limited. Additionally, while it may be argued that there may exist some task- and expertise-dependent features of QE, the central premise of QE training is the search for a *putative optimal behavior*, with QE times typically being averaged out across trials and participants (Dicks et al., 2017a). However, evidence is emerging that variability in gaze patterns in learning and performance are task- and individual-specific as are many movement behaviors. This observation highlights the fallacy of attempting to replicate a *universal optimal gaze pattern* to sit alongside optimal universal movement patterns (Dicks et al., 2017a).

In summary, research has shown inconclusive results for effects of brain training (Simons et al., 2016; Mirifar et al., 2017) and P-C training programmes and many questions remain. Nevertheless, more important to the understanding of sport performance, this process-oriented research has neglected the role of the body and environment in performance (Ring et al., 2015). The analysis of many P-C interventions, including QE training programmes, suffers the same methodological issues inherent in brain training studies: no pre-test baseline, no control group, lack of random assignment, passive control group, small samples, and lack of blinding when using subjective outcome measures (Simons et al., 2016; Walton et al., 2018). While these methodological weaknesses may be more apparent in brain training studies compared to P-C research, published evidence rarely shows zero effects of training interventions (null hypothesis is supported), implying universal benefits of these process training programmes. Further research is needed to understand whether the apparently universally successful outcomes of process training studies may actually be more indicative of Psychology’s problem with replication and publication bias more generally.

In order to consider how we can best develop P-C skills in performers, we need to undertake a critical review of the mechanisms and theory underpinning the current approaches used. We undertake this task next with a focus on Additive Models, the role of transfer, and the evaluation of the neuroscience underpinning P-C programmes.

## Additive Models of Learning

To examine efficacy of cognitive training programmes, such as generic computer-based brain training programmes or perceptual training programmes, we need to consider the rationale or theoretical beliefs about learning behind such approaches and then consider the empirical evidence. The basic assumption of this neurocomputational approach is that brain functions process input information and produce behavioral outputs like a computer (Anson et al., 2005). This approach favors the acquisition of knowledge indirectly through the enrichment of representations of the world in the brain. Therefore, a common approach adopted by applied sport psychologists is to provide knowledge about performance in the classroom or laboratory, before later (hopefully) applying it (Andersen, 2000; Weinberg and Gould, 2011). This approach is implicitly based on ideas from *formal discipline theory*, which has been the basis of education systems for centuries (Simons et al., 2016). This theory suggests that the mind consists of capacities (e.g., concentration, reasoning ability, memory) that can be improved through exercise, with the brain being just like a muscle that can be trained (Barnett and Ceci, 2002; Taatgen, 2013; Simons et al., 2016). Hence, each capacity can be developed generally, and in isolation from action in a performance environment, before being applied or transferred into practice in step-like sequences (Taatgen, 2013).

Despite empirical evidence suggesting that the development of a more generic knowledge base is limited, the additive, modular, step-like approach to learning key cognitive capacities supporting performance is strongly embedded in applied sport psychology. For example, Williams (1986; 2010) proposed a four-step model of integrating sport psychology techniques such as goal setting or relaxation into performance. Similar programmes were promoted by sport psychologists working for the National Coaching Foundation in the UK in the early 1980s. For example, it was believed that athletes could improve their concentration by utilizing “concentration grids” where they could find and cross off numbers 1–100 in a 10 × 10 numbered square (see https://cgridid.com/2017/04/03/concentration-grid-for-coaches-and-sports-psychologyperformance-professionals/ for a contemporary version) or learn progressive muscular relaxation techniques *via* an audiotape.

Despite recent potential advances in theoretical approaches to develop a more connected approach to movement analysis with “parts” being seen as more connected than in a traditional motor programming model (e.g. Hossner et al., 2015), in reality, the additive model is still strongly represented in practice design, for example, in the common part-whole approach to learning. In this approach, practitioners break a task down into its subcomponents to reputedly make learning easier. Decomposing a task into parts is purported to help develop greater performance consistency and stability (Handford, 2006). A proposed theoretical premise of this approach is motor programming (e.g. Schmidt, 1975), which, despite the emergence of contemporary neural computation theories of brain and behavior remains a prevailing theoretical model in motor control and learning (e.g., Shea and Wulf, 2005; Schmidt and Wrisberg, 2008; Summers and Anson, 2009). Hence, advocates of such approaches suggest that tasks composed of serially organized motor programmes are best suited to part-whole learning (Schmidt and Young, 1986). For example, tennis serving is proposed as a task where there is “clear evidence that practicing the subtasks in isolation can transfer to the total task” (Seymour, 1954 cited by Schmidt and Young, 1986, p. 23). Apparently, this is not surprising as the subtasks are essentially independent activities with little difference when performing them apart or whole. Accordingly, tennis serving is made up of two separate motor programmes (i.e., the ball-toss backswing as the first programme and the programme which produces the hit) that run sequentially (Schmidt and Young, 1986). However, there is limited neuroscientific evidence in support of this explanation, with empirical research questioning the efficacy of additive approaches in skill acquisition. A number of studies have shown that breaking actions down to improve modules or subphases does not lead to transfer when performing the whole task. For example, in tasks such as tennis or volleyball serving, coaching manuals have followed the model of part-whole learning emphasizing that a consistent ball toss is crucial to the success of the serve (Davids et al., 2001a). Coaching practice, therefore, focuses on developing a stereotyped toss action in isolation from the “hit.” Commonly, coaches put a small hoop or draw a chalk circle on the court surface and require players to throw the ball up to land inside the hoop. Only when consistency is achieved do coaches “add in” the hitting component. However, evidence shows that even expert tennis and volleyball players do not actually achieve invariant positioning in the vertical, forward-back, and side-to-side toss of the ball. Handford (2006) observed senior international volleyball players and found that the only invariant feature of their serves was the vertical component of the toss, with the forward-back and side-to-side dimension showing high levels of variability. It seems that servers aim to create temporal stability between the time of peak height of the ball toss and the time required for the forward swing of the hand to contact the ball. In a study to compare ball toss characteristics in part and whole tasks, the variability of the peak height of ball toss, when undertaking part practice, and the mean value for peak height was much greater than when the whole task was performed (Handford, 2006). Decomposing the task led to movement patterns that were dysfunctional for performance, and the key to skill acquisition was to learn to couple perception and action (interrupted by part training methodology). Other evidence questioning the usefulness of decomposing complex motor skills into smaller parts in actions that require individuals to couple their movements to the environment to achieve task goals exists in research on locomotor pointing tasks such as long jumping or cricket bowling. A nested task attached to the end of a run-up like jumping, or throwing an implement or ball, emphasizes the importance of the run-up to achieve a functional position to successfully complete the added task. Unfortunately, this emphasis has led to some coaches focusing on developing a stereotyped run-up. For example, in the long jump, athletes are asked to practice “run-throughs” without the need for jumping. However, empirical evidence has highlighted differences in gait regulation strategies when there is a requirement to jump rather than simply run through the pit (Glize and Laurent, 1997). Motor programming models of skill performance have had a significant impact on coaching of run-ups. For example, the belief that run-ups can be simply “run-off” with no need to engage with the environment is seen in the advice of former fast-bowling great and coaching guru, Dennis Lillee (Lillee and Brayshaw, 1977). Lillee suggests that the bowler who is having no-ball problems should simply put down a marker on the outfield, close his (or her) eyes, and run-up to “bowl” and mark the point at which the ball is delivered. After a few trials, the bowler will “know” the ideal run-up length, which should be measured and transferred to the game. Consequently, it is now common to observe cricket bowlers calibrate their run-ups with a tape measure. However, empirical evidence again rejects the idea of stereotyping of foot placement, reporting refined adaptations of gait, regulated by informational constraints of the environment, most commonly picked up by vision (de Rugy et al., 2002). In fact, continuous perception-action coupling during human locomotor pointing (i.e., running to place a foot on a target) has been demonstrated by athletes who make adjustments to their foot positioning as and when needed throughout the entire run-up (Renshaw and Davids, 2004). Continuous gait adjustments were found to be based on perception of the athletes’ current versus requisite positioning of the foot in relation to a target (Renshaw and Davids, 2004). Some expert coaches are aware of this concept and have noted that the ability to perceive the difference between current and ideal footfall positioning evolves through practice and experience and is part of the skill set of elite athletes (Greenwood et al., 2012).

In summary, evidence in support of additive models is somewhat flawed, and even studies of what might be viewed as highly “repeatable techniques,” such as running (Kiely, 2017), have highlighted that even when expert runners run at steady paces, movement patterns continuously vary. In fact, a key property of human movement systems, degeneracy (i.e., the emergent organization of the movement system in many different ways to achieve the same outcome), promotes efficiency and robustness in performance. When systems display increased stability and reduced complexity, for example, due to wear and tear due to chronic injury, misuse, or disuse, it can lead to performance decrements and further injuries (Kiely, 2017).

## Transfer

In elite sport, where time is precious, planned activities need to be empirically supported by evidence. An essential question for sport psychologists working with sports organizations is Do indirect methods of learning transfer to actual task performance? Practitioners and sport psychologists need to have confidence that prior experiences will prepare participants for novel situations and that practicing one task will improve performance of a related task. The rest of this paper will focus on the question of how much trust can be placed on perceptual-cognitive research and training activities undertaken *via* computer training or in laboratories or classrooms. How effective are these methods in contributing to improve cognition, perception, and action in performance settings? Here, we focus on the key issue: transfer.

The concept of transfer is central to the discussion of effectiveness of perceptual-cognitive training programmes in enhancing sport performance. Transfer of learning has been defined as “the gain (or loss) in the capability for responding in one task (termed the criterion task) as a function of practice or experience in some other task(s)” (Schmidt and Young, 1986, p. 2). Despite the prevalence of ideas from *formal discipline theory* in contemporary sport psychology, opposition to these ideas was initially raised by Thorndike (1922). Thorndike proposed the identical elements theory of transfer which argued that to transfer, elements of the practice task must be tightly coupled to the properties (stimuli, tasks and responses) in the performance task (Simons et al., 2016). Hence, only tasks with near transfer (i.e., those tasks which share common features) are likely to result in effective transfer, while far transfer (i.e., tasks/domains with significantly different common elements) is less likely to be effective. More recent models of skills acquisition have attempted to overcome the problems of explaining far transfer as per Thorndike’s theory by proposing models of skill acquisition such as the ACT production system (Newell, 1980; Anderson, 1982). Production models suggest that an initial stage of skill learning is characterized by the development of a declarative knowledge base (where a person initially learns only the “facts” about the skill), which is converted into procedural knowledge (Anderson, 1982). The procedural knowledge (or production phase) uses the declarative knowledge interpretively, with an initial composition of elements that takes sequential elements and collapses them into single complex production units (i.e., chunking-Chase and Simon, 1973). The procedural phase involves application of knowledge learned, meaning that nondomain-specific knowledge can be applied to perform in a specific domain, supporting behaviors appropriate to that domain (Anderson, 1982). While the ACT model was updated with proposed neuroscientific support in 2004 (Anderson et al., 2004), to our knowledge there has yet to be a sustained attempt to integrate the model into a practice programme in sport for training brain or P-C processes. It is apparent that, in production models, knowledge necessary for a particular task is encoded in a set of internalized rules in a “condition-action” paradigm (Taatgen, 2013). The result is that production models seek to explain how far transfer may occur by suggesting that the declarative knowledge base acts as the main source of transfer (Taatgen, 2013), suggesting the efficacy of domain-general cognitive abilities (Sala and Gobet, 2017).

But, a key issue is how to separate specific elements from general items in order to maximize transfer (Taatgen, 2013). What components are “near” and “far” in this model of transfer? There are other limitations in production models for explaining transfer, for example, What is the starting point of knowledge? Cognitive models therefore suffer from the problem of prior knowledge in some form (Taatgen, 2013). Finally, enhancement should not be mistaken with transfer (Moreau and Conway, 2014); enhancement is demonstrated when an experimental condition shows significant improvement in any kind of measurement task relative to the control condition; this is not the same as responding in one task (sport) as a function of practice in some other task (brain training task).

In summary, there is significant empirical evidence that practice only generally improves performance for a practiced task, or nearly identical ones, and does not greatly enhance other related skills. Generic noncontextual interventions may have limited value (Simons et al., 2016). The current view on transfer can be considered in terms of a continuum spectrum; the bigger the similarity between tasks, the bigger the transfer (Barnett and Ceci, 2002).

## Evidence from Neuroscience Relevant to Process Training

Given the arguments on transfer, it is clear that brain training programmes typically focus on performance during relatively general tasks (promoting at best far or general transfer). In line with the general discipline theory of learning, advocates for brain training claim that learning these skills by, for example, playing computer-based games will make them “smarter, more alert, and able to learn faster and better” (Lindenberger et al., 2017). That is, they will lead to the development of a more general range of skills in a wide range of contexts. However, while evidence is lacking for these claims (e.g., Sala et al., 2017), advocates for cognitive training programmes have turned to the science of neural plasticity to support their claims (Simons et al., 2016). Understanding how brain training might work requires a compelling theoretical rationale for explaining how and why processes in brain development and, in particular, the role of brain plasticity in adaptive learning. Without a comprehensive explanation one is left with an operational description of brain processes as modular which are assumed to be trainable in isolation. So what does the science actually tell us? Plasticity is defined as “the brain’s capacity to respond to experiences with structural changes that alter the behavioral repertoire” (Lindenberger et al., 2017, p. 261). It is a key feature of learning, remembering, and adapting to changing conditions of the body and the environment (Power and Schlaggar, 2017). When learning a new skill, studies of brain development have demonstrated that the mechanisms of plasticity can be modeled as a two-phase process, with an overproduction phase preceding a pruning phase (Lindenberger et al., 2017). The increase in the number of synapses at the beginning of the plastic episode corresponds with an initial exploration phase as the learner searches for a functional task solution (Chow et al., 2015). Once found, stabilization occurs, with connections that “work” being selected and nonfunctional neural patterns decaying. Consequently, changes in brain gray matter volume are specific to the experiences undertaken with the brain exhibiting “dramatic, larger scale changes in organization in response to experience” (Power and Schlaggar, 2017, p. 4). This point has important implications for learning and practice design highlighting the need for careful thought to promote functional neural organization. for example, neuroimaging of musicians who play stringed instruments revealed larger than normal sensory activation in the cortex for the fingers specifically involved in string manipulation (i.e., the left digits), but not for the thumb (which is not used) (Power and Schlaggar, 2017).

Until recently, brain plasticity was viewed as being particular prominent for brief critical periods or “windows of opportunity” early in life. The long-held view of critical windows has been challenged by recent advances in understanding brain development, which has revealed that brain plasticity occurs throughout the lifespan. This “new” understanding has led to great interest in potential interventions that could reverse age-related decrements in cognitive functioning (Power and Schlaggar, 2017).

There is potential to exploit inherent neuroplasticity for those interested in brain training, such as sport practitioners and psychologists working with adults who may wish to change dysfunctional movement patterns (e.g., an errant golf swing or basketball shooting technique). Could a deep, stable attractor (i.e., pattern) be linked to mechanisms of brain plasticity and to the closing off of critical periods? Changing action when a movement pattern is well established is notoriously difficult and perhaps relates to the idea of the closing off of critical periods which may involve the physical stabilization of synapses and network structure by myelin (a fatty substance wrapped around the axons of neuron, providing insulation and increasing the speed of neural conduction). Given the formation of new neural connections is metabolically costly (Lindenberger et al., 2017), closing off critical periods would make sense. A potentially useful strategy may be to exploit established attractors such as walking patterns (for different forms of bipedal locomotion) or well-learned implement swinging actions to explore other object-striking tasks. Perturbing a stable attractor could be viewed of sufficient importance and have some evolutionary (in performance terms) value. Consider, for example, the challenge of neural reorganization after a stroke, when previously functional behaviors can become dysfunctional, the brain undergoes a dynamic process of reorganization and repair and behavior remodeling shaped by new experiences (Jones, 2017). Motor impairments *invite* adaptations for motor system with different characteristics, a process considered as “skill re-acquisition.” When previous ways of performing an action no longer work (due to impairment, conditions, or chronic injury), the process of adaptation involves skill refinement (including perception, action, and cognition), which is practice dependent. It quickly becomes apparent that there is no typical way of performing an action because of the personal constraints that each individual needs to satisfy during movement performance. For this reason, rehabilitation programmes need to focus on functionality, defined as successful task completion by each individual, depending on the uniqueness of his/her personal constraints (e.g., intact limbs, muscle wastage or damage, degradation of the nervous system through conditions like peripheral neuropathy, level of perceptual or cognitive impairment). Nervous system regenerative processes occur over long time spans (months or longer) but are particularly dynamic early (days to weeks) after a stroke (Jones, 2017), providing a critical window for skill reacquisition. It would appear that neurobiological reorganization mirrors early learning experiences with initial overproduction followed by pruning. There is a possibility that research findings on neural reorganization in stroke patients may have potential implications for practitioners who wish to change perception-action skills in unimpaired participants. Just like in a stroke, a breakdown in performance as a result of a disruption to existing functional patterns or connections within the CNS *demands* system reorganization in an attempt to develop functional behavior solutions to achieve desired outcomes (Alexandrov et al., 1993; Järvilehto, 2001). However, these experiences may compete with one another in shaping neural reorganization patterns, as in learning a novel task in unimpaired individuals (see Jones, 2017). The interaction between cognitions, perceptions, and actions to regain functionality is highlighted in these cases as system reorganization or skill reacquisition.

The previous sections have highlighted the limitations of current methodologies and mechanisms purported to support effects of P-C training on behavior change and refinement. Throughout, it is clear that a single focus on developing cognitive skills and knowledge situated inside the heads of individuals has led to interventions that are failing to achieve their goals, i.e., transfer of learned P-C skills is weak. There is a need for research and practice to be underpinned by a theoretical model that sets processes of cognition, perception, and action in an embodied world. Here, we propose that the transactional meta-theory of ecological dynamics is a candidate framework, emphasizing the continuous emerging relations between each individual and the environment during behavior, which can meet this requirement.

## An Ecological Dynamics Approach to Evaluating Relative Merits of Process Training Programs

Ecological dynamics can help in guiding researchers in gaining a deeper understanding of merits of perceptual-cognitive training, including “brain training” (Davids and Araújo, 2016). Ecological dynamics elucidates understanding of how perception, action, and cognition emerge from interacting constraints of performer, task, and environment (not solely from the individual, nor from component parts, like the brain). It focuses on the role of adaptive variability in skilled individuals perceiving affordances in performance environments (Araújo et al., 2017). For example, How is useful information revealed as such for an individual performing a given task? How can relevant contextual information be distinguished from irrelevant information, *before* the detected information is “transmitted” to the brain, as proposed in theories emphasizing the role of perceptual-cognitive processes? This is an important question because explanations of brain training effects rely, traditionally, on assumptions that the brain processes (detects, attends to, learns, or memorizes) “relevant” information. Information from a sport context will then “feed” neural networks, allowing brain structures to organize (programme) a motor response. But, how are “brain training” games designed to distinguish distracting informational sources in competition from those which are simply raising alertness for each individual?

From a neurocomputational view, the putative role of the brain is to attribute meaning to stimuli, process internal representations, and select an already programmed response. The problem is that the starting point is missing in a brain-centered explanatory framework: How is an action that helps the body to search for relevant information “programmed by the brain”? A process-oriented, representational explanation to this question requires a “loan on intelligence” (Dennett, 1991). One possible answer to such a challenging question implies a clear understanding of the role of constraints and task information in explaining how intertwined processes of perception, cognition, and action channel goal achievement in athletes (Araújo et al., 2017). And, this explanation cannot be confined to how task constraints and information are represented in the brain, because this will always postpone the answer to the question (require a loan on intelligence) concerning how these task constraints and information sources were selected in the first place.

An ecological dynamics framework that formally includes both the individual (body and brain) and the environment (task constraints) does not centralize the brain and its training as the sole explanation for expert performance, as implied in “brain training” claims. The view that visual information from monitors is sufficient to train the brain is too restricted from an ecological dynamics viewpoint. This advocates that there are more constraints than eye movements, brain waves, and button pressing in explaining and training for expert performance in sports (Davids et al., 2015). This is one reason why it may be timely for perceptual-cognitive training in general, and brain training research in particular, to focus on the role of interacting constraints. An interacting constraints model can be used to theoretically inform experiments and practice on behaviors and brain function. To explain that an expert performer is already “in the right place at the right time” and “reads the game well,” an ecological dynamics perspective can address how the brain needs to be understood beyond an “organismically biased” perspective (Davids and Araújo, 2010b). The separation of organism and environment leads to theorizing in which the most significant explanatory factors in behavior are located within the organism. The upshot is that causes for behavioral disturbances are equated with perturbations in brain function (e.g., Yarrow et al., 2009). This reductionist explanation of sport performance, as solely dependent on “brain” processes, seems to endorse psychological attributes (representations, programmes, schemas, scripts) as specific anatomical substrates, rather than emerging from continuous interactions of the *individual-environment system*. Analysis of a “brain-centered” perspective reveals a belief that the brain perceives, executes, conceives, represents, and constructs an action and not the organism-environment system. For this reason, some neuroscientists have argued that sport performance represents a valuable natural context for their research to address (Walsh, 2014). However, it is the whole individual, rather than separate anatomical parts of his/her body, who perceives and acts during dynamical interactions with sport environments (Araújo and Kirlik, 2008). Performance is not possessed by the brain of the performer, but rather it can be captured as a dynamically varying relationship that has emerged between the constraints imposed by the environment and the capabilities of a performer (Araújo and Davids, 2011).

From an ecological dynamics perspective, current research on brain training and neurofeedback raises questions such as: How does a given value of quiet eye relate to emergent coordination tendencies of an individual athlete as he or she attempts to satisfy changing task constraints? How do skilled performers adapt and vary brain wave parameters during performance to support coordination of their actions with important environmental events, objects, surfaces, and significant others? Rather than looking for optimal values of brain waves or quiet eye, it would be more important to look for “critical threshold bandwidths” which could be functionally distinctive according to task and individual constraints, within and between expertise levels, while studying emergent actions in sport performance (Davids and Araújo, 2016).

From an ecological dynamics approach, behavior can be understood as self-organized, in contrast to organization being imposed from inside (e.g., the brain) or outside (e.g., the instructions of a videogame). Performance is not prescribed by internal or external structures, yet within existing constraints, there are typically a limited number of stable solutions that can achieve a desired outcome (Araújo et al., 2017). From an athlete’s point of view, the task is to exploit physical (e.g., rule-determined playing area characteristics) and informational (e.g., movements of other players) constraints to stabilize performance behaviors. Constraints have the effect of reducing the number of configurations available to an athlete at any instance, signifying that, in a performance environment, behavior patterns emerge under constraints as less functional states of organization are dissipated. Athletes can exploit this tendency to enhance their adaptability and even to maintain performance stability under perturbations from the environment. Importantly, changes in performance constraints can lead a system towards bifurcation points where choices emerge as more specific task information becomes available, constraining the environment-athlete system to switch to a more functional path of behavior (Araújo et al., 2006). Of significance for this discussion, neuroplastic changes induced by sport practice are more long-lasting when practice is self-motivated rather than forced by a decontextualized imposed task (Farmer et al., 2004).

In ecological dynamics, all parts of the system (brain, body, and environment) are dynamically integrated during action regulation (see also Moreau and Conway, 2014, Moreau et al., 2015). As a starting point, the concepts of affordances, self-organization, and emergent behaviors make it likely to expect that there may be functional variability in brain functioning characteristics (within critical bandwidths) among athletes as they perceive affordances under different task constraints. Seeking optimal values of brain processes, due to training with digital devices, is rather limited to more general effects with currently unknown transfer effects to performance environments.

## Conclusions

Elite sports organizations often spend significant time and money on off-field activities designed to build knowledge and train processes to give them the extra “one percent” and a “crucial edge” on their rivals. How effective and efficient is the use of valuable resources on process training activities in elite sport? Do these process training programmes work and, if so, how can we make them even better?

In this paper, we argued that the term “process training” captures activities and methodologies, which are predicated on assumptions that perceptual and cognitive systems and brain processes can be trained in isolation from the informational constraints of competitive performance environments. For this reason, process training, in general, can be critically evaluated for its effectiveness and efficient use of time and money in achieving performance outcomes. Current research suggests that process training has little evidence to support effectiveness and efficiency with respect to performance behaviors (e.g., see Harris et al., 2018).

Compelling evidence exists that the dominant process training methodologies tend to be operationally defined on the basis of an assumption of modularized subsystems and lack a clear theoretical rationale to underpin their effective *implementation* in elite training programs. These suggestions are in line with arguments of Simons et al. (2016, p. 161), when discussing the value of brain training. They suggested that “in order to provide effective guidance…we need assessments of the effectiveness of the training itself, but we also need studies assessing the comparative effectiveness of interventions that do work. Moreover, we need to consider the opportunity costs [including time demands] and the generalizability of those interventions. At present, none of those further analyses are possible given the published literature.” They further added that “cognitive-intervention research needs more complete translational theories that meaningfully connect lab based measures to objective measures of everyday performance (p. 161)”.

In this position paper, we considered theory and evidence to determine the effectiveness of current indirect methods of developing the underlying neuropsychological mechanisms of sports expertise. We highlighted the focus of P-C training on modular cognitive and perceptual structures in the majority of studies, discussing insights on limitations of P-C training. In line with ideas of Broadbent et al. (2015), we concluded that the current evidence that P-C training methods leads to effective transfer to performance is limited and requires more work. A key proposal here is that any P-C training programme claimed to have a positive impact on performance must be *representative* of performance environments, resulting in *fidelity* of response actions (Travassos et al., 2013). Current P-C training is hamstrung by the decision of sport psychologists to underpin interventions with traditional cognitive and experimental psychological process-oriented perspectives. This theoretical rationale leads to a biased modularized focus on the organism and a glaring neglect of environmental constraints on behavior (Araújo and Davids, 2011). The biased emphasis on acquisition of enriched internal representations typically fails to acknowledge (and embrace) the dynamic interdependence of knowledge, emotions, and intentions at the heart of mutually constraining perception-action couplings that underpin performance. A problem is the advocacy of key concepts and ideas of formal discipline theory where psychological process modules are trained (like muscles) in isolation before being applied in practice. We discussed the relatively weak empirical evidence that supports this approach. We exemplified this lack of empirical support by focusing on part-whole learning in the context of Schmidt’s (1975) schema theory and Thorndike’s (1922) identical elements theory and contemporary iterations such as Anderson’s (1982) ACT theory. We concluded that there are limitations in production models for explaining transfer, for example, by highlighting that performance enhancement should not be mistaken for transfer (Moreau and Conway, 2014). The latter may only be demonstrated when significant improvement in one task (sport) can be shown to be a function of practice in some other task (brain training task), which is currently lacking in evidence.

The putative mechanisms underpinning P-C training requires researchers to evaluate evidence of neuroplasticity and brain development. In this respect, it is important to note how current thinking has moved away from critical periods or windows of opportunity to develop P-C skills to a more lifelong view of neuroplasticity. Overall, the neuroscience evidence in support of P-C training is harmonious with experimental findings from P-C studies showing that functional neural connectivity is specific to the experiences undertaken. The result is that changes in the brain exhibit “dramatic, larger scale changes in organization in response to experience” (Power and Schlaggar, 2017).

So how can current research help us enhance P-C training programmes? Here, we proposed that adopting an ecological dynamics perspective may help researchers to frame interventions to enhance understanding of continuous, complex interactions between individual and team P-C skills from a brain-body-environment relationship (Gibson, 1979; Chemero, 2003; Kiverstein and Miller, 2015). Central to this approach is a focus on ensuring that individual-environment mutuality sits at the heart of any intervention design. Sampling of the environment (e.g., Brunswik, 1956; Pinder et al., 2011a), when designing interventions to enhance P-C skills, has been largely neglected. Consequently, it has yet to be established *if* or *how* perceptual mechanisms such as QE can inform the design of practice environments for the purpose of skill development. Ecological dynamics and its emphasis on the integrative, inter-connected relationship between cognitions, emotions, intentions, and emergent perception-action couplings posit a complementary role for indirect and direct methods of learning P-C skills. Adopting such integrative approaches moves the field beyond the unhelpful cognitive versus ecological debate and takes an embodied view of cognition allowing researchers and practitioners to begin to design-in factors such as context specific knowledge and their link to intentions, perceptions, and actions.

In summary, we have attempted to draw on theoretical insights that can better articulate cognition, perception, and action as it relates to the dynamic performance environment inhabited by experts, rather than the stale and contrived research “tests” performed in computers in laboratories. There are clear epistemological and methodological conflicts here that require a reimagined breadth of methodology for P-C training to be utilized beyond the pages of academic journals. Research methodologies must cater for the ambiguity of multiple acting constraints upon the performance environment. A research approach grounded in the theory of ED has the potential to provide a powerful theoretical rationale for how to develop P-C and brain processes in expert performers by designing dynamic training tasks which call for intertwined cognition, perception, and actions. This focus will ensure that performers can develop adaptive variability demonstrated by skilled individuals when perceiving affordances in performance environments (Araújo et al., 2017). Accordingly, P-C training should be understood as a process by which athletes become attuned to action-specifying sources of information. Future studies in P-C training need to be grounded in a theoretical model whose methodologies support tasks with representative design, furthering the coupling of perceptual attunement and skill acquisition.

## Author Contributions

IR co-created the paper and led the writing of the paper. KD and DA co-created the paper and made a significant contribution to the writing. AL contributed to the sections on P-C training. WR, DN, and BF contributed to the section on Quiet Eye and the summary.

### Conflict of Interest Statement

The authors declare that the research was conducted in the absence of any commercial or financial relationships that could be construed as a potential conflict of interest.
